# Flexible Roles for American Indian Elders in Community-Based Participatory Research

**DOI:** 10.5888/pcd13.150575

**Published:** 2016-06-02

**Authors:** Shannon Whitewater, Kerstin M. Reinschmidt, Carmella Kahn, Agnes Attakai, Nicolette I. Teufel-Shone

**Affiliations:** Author Affiliations: Kerstin M. Reinschmidt, Carmella Kahn, Agnes Attakai, Nicolette I. Teufel-Shone, University of Arizona, Tucson, Arizona.

## Abstract

Community-based participatory research builds partnerships between communities and academic researchers to engage in research design, decision making, data collection, and dissemination of health promotion initiatives. Community-based participatory projects often have formal agreements or defined roles for community and academic partners. Our project (November 2012–November 2014) was designed to document life narratives of urban American Indian elders as a foundation for developing a resilience-based health promotion curriculum for urban American Indian adolescents aged 12 to 18. We used a flexible method for engaging community partners that honored the individual strengths of elders, encouraged them to describe how they wanted to contribute to the project, and provided multiple ways for elders to engage with university partners. We invited elders to participate in one or more of the following roles: as members of consensus panels to develop interview questions, as members of a community advisory board, or as participants in individual qualitative interviews. The flexibility of roles gave elders the opportunity to serve as advisors, co-developers, interviewees, or reviewers during 2 years of curriculum development. Engaging American Indian elders in the research process acknowledged the multiple layers of expertise they had as traditional leaders in the community while promoting trust in and ownership of the project. This flexible technique can be used by other communities that may not be comfortable with structured processes of engagement.

## Introduction

Community-based participatory research (CBPR) can be used to promote health and conduct prevention research that fits the needs and culture of the community by engaging community members, community organizations, and researchers in all aspects of the research process (1,2). CBPR builds on the knowledge and experiences of community members to increase local participation, promote social change, and lead to sustainable health initiatives (3). CBPR is frequently used in public health, heralded as particularly useful in building partnerships that engage community and academic partners in research design, decision making, data collection, and dissemination of health promotion initiatives. Some researchers offering guidance and insight into the CBPR process recommend that community and academic leaders develop rules and operating procedures to formalize collaboration and facilitate partnerships (3,4). A structured approach with clear operating procedures is suggested to add transparency and establish expectations and roles of all partners; the process can include formal agreements and defined roles for participants (3,5).

Trust is an essential component in a successful collaboration, but it can be difficult to obtain because of negative perceptions about research in communities that were marginalized or received few direct benefits from research findings (3,6). During the past 10 years, CBPR-guided approaches have been well received by American Indian communities because of the emphasis on the equitable involvement of community members and local organizations throughout the research process (7–9). During a 2-year project, researchers from the Center for American Indian Resilience at the Mel and Enid Zuckerman College of Public Health at the University of Arizona partnered with the Tucson Indian Center, which provides social services to the urban American Indian community in Tucson, Arizona, to design and complete a research project that documented life narratives of urban American Indian elders to guide the development of a resilience-based curriculum for adolescents aged 12 to 18 (10,11). In this project, we offered a flexible approach to CBPR partnership that may be applicable in other communities uncomfortable with structured processes for engaging community partners. The plans for this research and associated activities were approved by the University of Arizona institutional review board and the Tucson Indian Center board of directors.

## Engaging American Indian Elders in Community-Based Participatory Research

In American Indian cultures, elders are valued as protectors, mentors, teachers, keepers of wisdom, and intergenerational transmitters of cultural knowledge (12). American Indian elders are often leaders in their communities and are expected to be involved in decision making (13). Urban American Indian elders, experienced in balancing traditional and contemporary living, were vital participants, partners, and advisors for this project. They share an American Indian worldview that forms the foundation of beliefs and values shaping the identity and behaviors of American Indian people (10).

In a departure from CBPR best practices (5,14), our project used a flexible approach in engaging American Indian elders as community partners. Without using formal agreements that defined time commitments or specific roles, we provided multiple opportunities for American Indian elders to engage in the project (Figure). The lack of defined roles for engagement allowed elders to decide how they wanted to participate: as members of consensus panels, as members of a community advisory board (CAB), or as individual interviewees. The absence of established roles and formal agreements for participation allowed elders to increase or decrease their engagement as they saw fit. This informal environment encouraged a more organic engagement than would otherwise have been possible, while respecting the cultural roles of American Indian elders.

**Figure:**
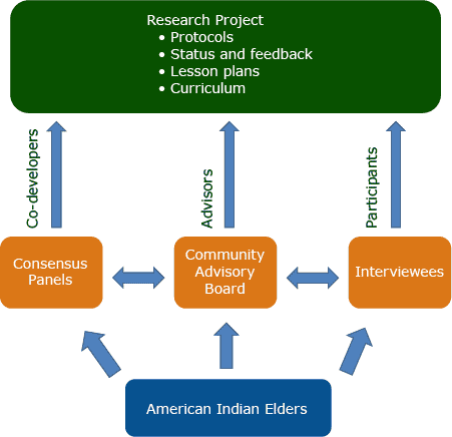
Self-selected roles of American Indian elders in a community-based participatory research project, Tucson, Arizona, 2012–2014.

The Tucson Indian Center provides monthly luncheons for American Indians elders aged 55 or older; these luncheons bring elders together to socialize with their peers and listen to presentations from various local organizations and programs. We used this natural platform to recruit elders for participation in consensus panels and interviews. Recruitment through verbal announcements at the monthly luncheons allowed the team of investigators to answer questions about the purpose of the project, to develop a relationship with the elders, and to form a purposeful sample of participants (15).

## Roles of Elders in Research Project

### Co-developers: consensus panels members

To generate discussion on the design of the interview guide and the content of the curriculum, we facilitated 4 consensus panels (16). Each panel met once for approximately 1 to 1.5 hours; the first panel met in March 2013; the second, December 2013, the third, January 2014, and the fourth, November 2014. The panels consisted of up to 22 elders. Besides self-identification as an American Indian elder and willingness to consent, we had no guidelines for participation in the consensus panels.

The absence of defined roles and formal agreements, such as time or participation commitments, contributed to the flexibility of the consensus panels. Included in this flexibility was the opportunity for the American Indian elders to choose to participate regardless of their experience or level of familiarity with the project. To acknowledge their time and show our appreciation for their participation, each time a community member participated in a consensus panel he or she was given a $20 gift card. Participation in the consensus panels provided elders with a chance to embrace the role of a co-developer in a community health initiative.

**The first consensus panel**. This panel reviewed a draft of our qualitative interview guide for cultural sensitivity, word selection, and clarity of the questions. Additionally, the elders requested that we follow cultural protocol and add an introduction for both the interviewer and the interviewee to establish rapport. After reaching a final consensus on its cultural appropriateness, the resulting interview guide was expanded from 11 questions to 25 questions to obtain information from interviewees on their experiences of historical trauma and resilience.

**The second consensus panel**. The goal of this panel was to review proposed lesson plans before the development of the curriculum. During this panel, elders were provided with a hard copy of proposed lesson plans. The investigators facilitated discussion about the plans and associated activities until the elders reached consensus.

**The third and fourth consensus panels**. These panels reviewed a draft of the resilience-based curriculum with particular attention to cultural sensitivity, missing information, and areas to strengthen. The elders critiqued compilations of video-recorded interviews and reviewed content of the individual lesson plans and the final curriculum. During the curriculum design and dissemination phases, elders members of these consensus panels bridged the gap between our usual methods of gathering qualitative data and the methods that are best suited to the American Indian community, thus increasing the likelihood of relevance of the curriculum for urban American Indian adolescents (3,6).

### Advisors: community advisory board members

In CBPR projects, a CAB is a structured entity that comprises selected community stakeholders and is typically created as a liaison to strengthen collaboration between the community and academic institutions (5,14). In most projects, CAB members are recruited and screened according to their expertise and resources with the objectives of 1) establishing a roadmap to partnership, 2) providing perspective and balance in partnerships and community health priorities, and 3) providing contacts and strategies (14,17). In addition to selection according to expertise, CAB members are often screened to ensure that the board consists of members who are bridge builders, “bringers” (people who bring resources to the project), and historians (3).

In January 2013, we began to recruit members for the CAB, which consisted of 19 members, including Tucson Indian Center staff, elders from the community, and researchers (11); the CAB met for the first time in April 2013. In contrast to projects that have recruitment criteria and screening processes for board membership, we allowed American Indian elders to self-select as CAB members without our requesting signed documents (3,14). We did not know the elders’ backgrounds or the experiences they would bring to the advisory board but trusted that their individual strengths and American Indian worldviews would collectively be an asset to the project.

To recruit for CAB members, the first CAB meeting was announced at the end of the first consensus panel. The names and contact information of elders willing to join the CAB were documented. The elders were then called and informed of the date, time, and location of CAB meetings as they were scheduled. CAB meetings occurred once every 4 to 6 weeks throughout the project, and although elders were informed of the meetings, they were not required to attend. Six out of 19 elders consistently attended CAB meetings; this consistency contributed to the project’s trust and partnership building (3).

During CAB meetings, CAB members were updated on the status of the project and also reviewed our research project outline and methods and provided feedback. Communication between the community and academic partners was emphasized by following cultural protocol and opening each meeting with a proper introduction to build trust. As CAB members, elders contributed to the cultural humility of our project by informing respectful and culturally sensitive research protocols (18). To retain and acknowledge their engagement, CAB members were provided with nonmonetary gifts for attendance at CAB meetings and opportunities for additional training to enhance their research skills. Such opportunities included training in data analysis and support for elder projects not related to this research. Two elders from the CAB elected to participate in qualitative research training to assist with data analysis for this project and also received Collaborative Institutional Training Initiative (CITI) Certification for Human Subjects Protection (https://www.citiprogram.org/).

### Participants: one-on-one interviews

We engaged 13 American Indian elders as participants in one-on-one interviews to document their life narratives. American Indian elders were asked to share their life narratives or personal stories of historical trauma, personal challenges, and strategies of resilience. Unlike the consensus panels, this aspect of elder project engagement had several eligibility criteria. Participants had to be self-identified as American Indian elders, aged 55 or older, living in Tucson, and willing to provide verbal and written consent to being interviewed. Elders who expressed interest in participating in the interviews were asked for their contact information, were contacted to set up interview times and location, and were provided with a $20 gift card at the completion of each interview. Through their storytelling, elders offered ideas for key themes for the curriculum. After being interviewed, elders were eligible to shift into being CAB members or consensus panel participants, or they could opt out of the process at any time.

**Reviews of personal interviews.** Shortly after providing their life narratives, the 13 elders were given an edited copy of their full interview and were asked to review it for additional editing. Two to three weeks later, elders met with a researcher, who used a semistructured interview guide, to discuss what they liked or disliked about their interview. Although most elders approved of their interviews and asked for no changes, one elder requested to redo his interview and another elder asked to change or remove pieces of his interview before they were incorporated into the final curriculum. Reviewing their recorded personal interviews offered the elders the opportunity to reflect on their own experiences of historical trauma and resilience. Sharing their own lived experiences for translation into a health promotion curriculum for adolescents helped promote ownership in the project (18).

## Discussion

During our 2-year project, we provided multiple opportunities for American Indian elders to participate without having to adhere to the formalized rules and operational procedures that are often recommended for CBPR projects to control the effectiveness and mixture of participants and to ensure that knowledge is transferred from the research to the field (3,4,14). The flexibility of elder engagement throughout this project gave elders the chance to self-select as members of a consensus panel, as members of a CAB, or as participants in one-on-one interviews. These opportunities provided a way of engaging American Indian elders throughout various points in the project and further cultivated the community–academic partnership (14,17). Although these opportunities for elder engagement are common in CBPR projects, our technique differed in that it allowed elders to self-select how and when they wanted to participate. This approach of self-selection resulted in a heterogeneous mix of participants with various backgrounds and strengths that honored the collaborative nature of CBPR (19). Self-selection also provided an element of empowerment for the elders who were engaged in roles of leaders, knowledge keepers, and knowledge transmitters. The elders’ participation allowed them to share their expertise in various roles of engagement at their choosing.

Our flexible approach to CBPR had several benefits, including various levels of elder involvement throughout the phases of the project; enhanced sense of elder ownership in the project; feedback from each consensus panel, the CAB, and the interviews on the interview process and curriculum development. We identified 2 major challenges: fluctuating attendance of participants at CAB meetings and lack of participation by some quiet elders at the consensus panels and CAB meetings.

### Lessons learned

**Privacy concerns.** Because all recruitment took place at the Tucson Indian Center, our research protocol addressed privacy concerns. Any elder who wished to participate in an interview was provided the option to be video recorded, audio recorded, or be completely anonymous. During an anonymous interview, notes were taken, but the elder’s voice was not captured and any markers indicating identity, such as names, were removed.

**Methods for active engagement.** To increase engagement, we offered gift cards as compensation for participating in consensus panels and interviews. However, other methods for active engagement were also used, including offering snacks and beverages at every meeting and offering nonmonetary gifts such as socks and puzzles. We also found it helpful to schedule consensus panels and CAB meetings directly after monthly elder luncheons because it relieved participants of any extra travel. 

**Sufficient representation.** Throughout this project, we recruited elders to participate in consensus panels and CAB meetings from the monthly elders’ luncheons. To ensure sufficient representation in the absence of regularly engaged elders, we provided frequent and thorough updates at every consensus panel and CAB meeting and occasionally at the elders’ luncheons. We also supported sufficient representation by directly asking quiet elders to share their input when we noticed that the most outspoken elders were the ones who volunteered the most feedback.

**Informal CAB.** Although best practices suggest formal agreement to support efficiency during the research process, we did not ask elders to sign a written commitment to attend regular CAB meetings. Although we did ask for verbal commitment at the beginning of the research process, we decided that it was not appropriate to enforce a signed commitment. The elders chose to participate according to their personal schedules, family engagements, and transportation needs. Because the CAB was flexible, elders could join at any time; this policy allowed for better representation but presented a challenge in interpreting feedback.

**Interpreting feedback.** Because we had no formal agreements for attendance or participation in any of the consensus panels or CAB meetings, attendance fluctuated. This fluctuation in attendance made consistent feedback difficult because one elder would suggest a particular change at one meeting, the change would be incorporated into the program, but at a later meeting, the change would be challenged by an elder not present at the earlier meeting. To ensure that we interpreted the feedback correctly, we provided a thorough update and progress report at the beginning of every meeting. Although the process was repetitive, it helped all participants, even those attending regularly.

The involvement of self-selected American Indian elders in every aspect of our research may provide evidence that using flexible techniques without defined roles for engagement and not having formal agreements benefitted this project. Engaging American Indian elders in this project acknowledged the traditional role of the American Indian elders as leaders, mentors, and teachers, which facilitated transparency that formed trust and gained community buy-in (10). This flexible, less-formal CBPR approach may be applicable for other urban American Indian communities that are not comfortable with the imposed structure of traditional research methods. It would also be worthwhile to explore the applicability of our more flexible CBPR approach in the context of reservation settings with the engagement of formal tribal structures and processes.

## References

[1] Kamanda A , Embleton L , Ayuku D , Atwoli L , Gisore P , Ayaya S , Harnessing the power of the grassroots to conduct public health research in sub-Saharan Africa: a case study from western Kenya in the adaptation of community-based participatory research (CBPR) approaches. BMC Public Health 2013;13(1):91. 10.1186/1471-2458-13-91 23368931 PMC3564692

[2] Gibson JE , Flaspohler PD , Watts V . Engaging youth in bullying prevention through community-based participatory research. Fam Community Health 2015;38(1):120–30. 10.1097/FCH.0000000000000048 25423250

[3] Horowitz CR , Robinson M , Seifer S . Community-based participatory research from the margin to the mainstream: are researchers prepared? Circulation 2009;119(19):2633–42. 10.1161/CIRCULATIONAHA.107.729863 19451365 PMC2796448

[4] Cargo M , Mercer SL . The value and challenges of participatory research: strengthening its practice. Annu Rev Public Health 2008;29(1):325–50. 10.1146/annurev.publhealth.29.091307.083824 18173388

[5] D’Alonzo KT . Getting started in CBPR: lessons in building community partnerships for new researchers. Nurs Inq 2010;17(4):282–8. 10.1111/j.1440-1800.2010.00510.x 21059145 PMC3203531

[6] Grigg-Saito D , Och S , Liang S , Toof R , Silka L . Building on the strengths of a Cambodian refugee community through community-based outreach. Health Promot Pract 2008;9(4):415–25. 10.1177/1524839906292176 17494947

[7] Jumper-Reeves L , Dustman PA , Harthun ML , Kulis S , Brown EF . American Indian cultures: how CBPR illuminated intertribal cultural elements fundamental to an adaptation effort. Prev Sci 2014;15(4):547–56. 10.1007/s11121-012-0361-7 23412946 PMC3726553

[8] Lonczak HS , Thomas LR , Donovan D , Austin L , Sigo RL , Lawrence N ; Suquamish Tribe. Navigating the tide together: early collaboration between tribal and academic partners in a CBPR Study. Pimatisiwin 2013;11(3):395–409. 25356083 PMC4209702

[9] Teufel-Shone NI , Siyuja T , Watahomigie HJ , Irwin S . Community-based participatory research: conducting a formative assessment of factors that influence youth wellness in the Hualapai community. Am J Public Health 2006;96(9):1623–8. 10.2105/AJPH.2004.054254 16873759 PMC1551937

[10] Kahn C , Oré C , Teufel-Shone N , Attakai A , Reinschmidt KM . Urban American Indian elders’ resilience: sources of strength for building a healthy future for today’s youth. Am Indian Alsk Native Ment Health Res. Forthcoming. 10.5820/aian.2303.2016.117 PMC6047895 27383089

[11] Reinschmidt KM , Attakai A , Kahn C , Whitewater S , Teufel-Shone N . Urban American Indian Elders’ Narratives of Historical Trauma and Resilience Shaping a Stories of Resilience Model. Am Indian Alsk Native Ment Health Res. Forthcoming. 10.5820/aian.2304.2016.63 PMC6014738 27536898

[12] Garrett MT , Parrish M , Williams C , Grayshield L , Portman TA , Rivera ET , Invited commentary: fostering resilience among Native American youth through therapeutic intervention. J Youth Adolesc 2014;43(3):470–90. 10.1007/s10964-013-0020-8 24096529

[13] Rasmus SM . Indigenizing CBPR: evaluation of a community-based and participatory research process implementation of the Elluam Tungiinun (towards wellness) program in Alaska. Am J Community Psychol 2014;54(1-2):170–9. 10.1007/s10464-014-9653-3 24756887 PMC4119544

[14] Newman SD , Andrews JO , Magwood GS , Jenkins C , Cox MJ , Williamson DC . Community advisory boards in community-based participatory research: a synthesis of best processes. Prev Chronic Dis 2011;8(3):A70. 21477510 PMC3103575

[15] Patton MQ . Qualitative research and evaluation methods. 3rd edition. Thousand Oaks (CA): Sage Publications; 2002.

[16] Coreil J . Group interview methods in community health research. Med Anthropol 1995;16(3):193–210. 8643022 10.1080/01459740.1994.9966115

[17] Norris KC , Brusuelas R , Jones L , Miranda J , Duru OK , Mangione CM . Partnering with community-based organizations: an academic institution’s evolving perspective. Ethn Dis 2007;17(1, Suppl 1):S27–32. 17598314

[18] Flicker S , O’Campo P , Monchalin R , Thistle J , Worthington C , Masching R , Research done in “a good way”: the importance of Indigenous elder involvement in HIV community-based research. Am J Public Health 2015;105(6):1149–54. 10.2105/AJPH.2014.302522 25880963 PMC4431085

[19] McKenna SA , Iwasaki PG , Stewart T , Main DS . Key informants and community members in community-based participatory research: one is not like the other. Prog Community Health Partnersh 2011;5(4):387–97. 22616206

